# How to Construct a Mixed Methods Research Design

**DOI:** 10.1007/s11577-017-0454-1

**Published:** 2017-07-05

**Authors:** Judith Schoonenboom, R. Burke Johnson

**Affiliations:** 1 Institut für Bildungswissenschaft, Universität Wien, Sensengasse 3a, 1090 Wien, Austria; 2 Department of Professional Studies, University of South Alabama, UCOM 3700, 36688-0002 Mobile, AL USA

**Keywords:** Methods of social research, Mixed methods, Qualitative methods, Quantitative methods, Research design, Mixed methods design, Mixing purpose, Timing of mixing, Point of integration, Design complexity, Methoden der empirischen Sozialforschung, Mixed Methods, Qualitative Methoden, Quantitative Methoden, Forschungsdesign, Mixed Methods-Design, Anlass des Methoden-Mix, Zeitpunkt des Methoden-Mix, Grad der Methodenintegration, Komplexität des Forschungsdesigns

## Abstract

This article provides researchers with knowledge of how to design a high quality mixed methods research study. To design a mixed study, researchers must understand and carefully consider each of the dimensions of mixed methods design, and always keep an eye on the issue of validity. We explain the seven major design dimensions: purpose, theoretical drive, timing (simultaneity and dependency), point of integration, typological versus interactive design approaches, planned versus emergent design, and design complexity. There also are multiple secondary dimensions that need to be considered during the design process. We explain ten secondary dimensions of design to be considered for each research study. We also provide two case studies showing how the mixed designs were constructed.

## Zusammenfassung

Der Beitrag gibt einen Überblick darüber, wie das Forschungsdesign bei Mixed Methods-Studien angelegt sein sollte. Um ein Mixed Methods-Forschungsdesign aufzustellen, müssen Forschende sorgfältig alle Dimensionen von Methodenkombinationen abwägen und von Anfang an auf die Güte und damit verbundene etwaige Probleme achten. Wir erklären und diskutieren die für Forschungsdesigns relevanten sieben Dimensionen von Methodenkombinationen: Untersuchungsziel, Rolle von Theorie im Forschungsprozess, Timing (Simultanität und Abhängigkeit), Schnittstellen, an denen Integration stattfindet, systematische vs. interaktive Design-Ansätze, geplante vs. emergente Designs und Komplexität des Designs. Es gibt außerdem zahlreiche sekundäre Dimensionen, die bei der Aufstellung des Forschungsdesigns berücksichtigt werden müssen, von denen wir zehn erklären. Der Beitrag schließt mit zwei Fallbeispielen ab, anhand derer konkret gezeigt wird, wie Mixed Methods-Forschungsdesigns aufgestellt werden können.

## What is a mixed methods design?

This article addresses the process of selecting and constructing mixed methods research (MMR) designs. The word “design” has at least two distinct meanings in mixed methods research (Maxwell 2013). One meaning focuses on the process of design; in this meaning, design is often used as a verb. Someone can be engaged in *designing* a study (in German: “eine Studie konzipieren” or “eine Studie designen”). Another meaning is that of a product, namely the result of designing. The result of *designing* as a verb is a mixed methods *design* as a noun (in German: “das Forschungsdesign” or “Design”), as it has, for example, been described in a journal article. In mixed methods design, both meanings are relevant. To obtain a strong design as a product, one needs to carefully consider a number of rules for designing as an activity. Obeying these rules is not a guarantee of a strong design, but it does contribute to it. A mixed methods design is characterized by the combination of at least one qualitative and one quantitative research component. For the purpose of this article, we use the following definition of mixed methods research (Johnson et al. 2007, p. 123):

Mixed methods research is the type of research in which a researcher or team of researchers combines elements of qualitative and quantitative research approaches (e. g., use of qualitative and quantitative viewpoints, data collection, analysis, inference techniques) for the broad purposes of breadth and depth of understanding and corroboration.

Mixed methods research (“Mixed Methods” or “MM”) is the sibling of multimethod research (“Methodenkombination”) in which either solely multiple qualitative approaches or solely multiple quantitative approaches are combined.

In a commonly used mixed methods notation system (Morse 1991), the components are indicated as *qual* and *quan* (or QUAL and QUAN to emphasize primacy), respectively, for qualitative and quantitative research. As discussed below, plus (+) signs refer to concurrent implementation of components (“gleichzeitige Durchführung der Teilstudien” or “paralleles Mixed Methods-Design”) and arrows (→) refer to sequential implementation (“Sequenzielle Durchführung der Teilstudien” or “sequenzielles Mixed Methods-Design”) of components. Note that each research tradition receives an equal number of letters (four) in its abbreviation for equity. In this article, this notation system is used in some depth.

A mixed methods design as a product has several primary characteristics that should be considered during the design process. As shown in Table 1, the following primary design “dimensions” are emphasized in this article: purpose of mixing, theoretical drive, timing, point of integration, typological use, and degree of complexity. These characteristics are discussed below. We also provide some secondary dimensions to consider when constructing a mixed methods design (Johnson and Christensen 2017).

**Table 1:** List of Primary and Secondary Design Dimensions

*Primary Dimensions*	*Primäre Dimensionen*
1. Purpose	Untersuchungsziel
2. Theoretical drive	Rolle von Theorie im Forschungsprozess
3. Timing (simultaneity and dependence)	Timing (Simultanität und Abhängigkeit)
4. Point of integration	Schnittstellen, an denen Integration stattfindet (Integrations-Schnittstellen)
5. Typological vs. interactive design approach	Systematischer vs. interaktiver Design-Ansatz
6. Planned vs. emergent design	Geplante vs. emergente Designs
7. Complexity	Komplexität des Designs
*Secondary Dimensions:*	*Sekundäre Dimensionen*
1. Phenomenon	Untersuchungsgegenstand
2. Social scientific theory	Ertrag für die sozialwissenschaftliche Theorie (Theoretischer Ertrag)
3. Ideological drive	Praktische Relevanz
4. Combination of sampling methods	Kombinierte Stichprobenstrategien
5. Degree to which the research participants will be similar or different	Grad der (Un)Ähnlichkeit der Forschungsteilnehmenden
6. Degree to which the researchers on the research team will be similar or different	Grad der (Un)Ähnlichkeit der Forschenden
7. Type of implementation setting	Untersuchungskontext
8. Degree to which the methods similar or different	Grad der (Un)Ähnlichkeit der Untersuchungsmethoden
9. Validity criteria and strategies	Gütekriterien und -strategien
10. Full study vs. multiple studies	Einzelstudie vs. verschiedene Studien

On the basis of these dimensions, mixed methods designs can be classified into a mixed methods typology or taxonomy. In the mixed methods literature, various typologies of mixed methods designs have been proposed (for an overview see Creswell and Plano Clark 2011, p. 69–72).

## Purpose

The overall goal of mixed methods research, of combining qualitative and quantitative research components, is to expand and strengthen a study’s conclusions and, therefore, contribute to the published literature. In all studies, the use of mixed methods should contribute to answering one’s research questions.

Ultimately, mixed methods research is about heightened knowledge and validity. The design as a product should be of sufficient quality to achieve *multiple validities legitimation* (Johnson and Christensen 2017; Onwuegbuzie and Johnson 2006), which refers to the mixed methods research study meeting the relevant combination or set of quantitative, qualitative, and mixed methods validities in each research study.

Given this goal of answering the research question(s) with validity, a researcher can nevertheless have various reasons or purposes for wanting to strengthen the research study and its conclusions. Following is the first design dimension for one to consider when designing a study: Given the research question(s), what is the purpose of the mixed methods study?

A popular classification of purposes of mixed methods research was first introduced in 1989 by Greene, Caracelli, and Graham, based on an analysis of published mixed methods studies. This classification is still in use (Greene 2007). Greene et al. (1989, p. 259) distinguished the following five purposes for mixing in mixed methods research:

1. *Triangulation* seeks convergence, corroboration, correspondence of results from different methods;

2. *Complementarity* seeks elaboration, enhancement, illustration, clarification of the results from one method with the results from the other method;

3. *Development* seeks to use the results from one method to help develop or inform the other method, where development is broadly construed to include sampling and implementation, as well as measurement decisions;

4. *Initiation* seeks the discovery of paradox and contradiction, new perspectives of frameworks, the recasting of questions or results from one method with questions or results from the other method;

5. *Expansion* seeks to extend the breadth and range of inquiry by using different methods for different inquiry components.

In the past 28 years, this classification has been supplemented by several others. On the basis of a review of the reasons for combining qualitative and quantitative research mentioned by the authors of mixed methods studies, Bryman (2006) formulated a list of more concrete rationales for performing mixed methods research (see Appendix). Bryman’s classification breaks down Greene et al.’s (1989) categories into several aspects, and he adds a number of additional aspects, such as the following:

(a) *Credibility *– refers to suggestions that employing both approaches enhances the integrity of findings.

(b) *Context *– refers to cases in which the combination is justified in terms of qualitative research providing contextual understanding coupled with either generalizable, externally valid findings or broad relationships among variables uncovered through a survey.

(c) *Illustration *– refers to the use of qualitative data to illustrate quantitative findings, often referred to as putting “meat on the bones” of “dry” quantitative findings.

(d) *Utility *or improving the usefulness of findings – refers to a suggestion, which is more likely to be prominent among articles with an applied focus, that combining the two approaches will be more useful to practitioners and others.

(e) *Confirm and discover *– this entails using qualitative data to generate hypotheses and using quantitative research to test them within a single project.

(f) *Diversity of views *– this includes two slightly different rationales – namely, combining researchers’ and participants’ perspectives through quantitative and qualitative research respectively, and uncovering relationships between variables through quantitative research while also revealing meanings among research participants through qualitative research. (Bryman, p. 106)

Views can be diverse (f) in various ways. Some examples of mixed methods design that include a diversity of views are:

- Iteratively/sequentially connecting local/idiographic knowledge with national/general/nomothetic knowledge;
- Learning from different perspectives on teams and in the field and literature;
- Achieving multiple participation, social justice, and action;
- Determining what works for whom and the relevance/importance of context;
- Producing interdisciplinary substantive theory, including/comparing multiple perspectives and data regarding a phenomenon;
- Juxtaposition-dialogue/comparison-synthesis;
- Breaking down binaries/dualisms (some of both);
- Explaining interaction between/among natural and human systems;
- Explaining complexity.

The number of possible purposes for mixing is very large and is increasing; hence, it is not possible to provide an exhaustive list. Greene et al.’s (1989) purposes, Bryman’s (2006) rationales, and our examples of a diversity of views were formulated as classifications on the basis of examination of many existing research studies. They indicate how the qualitative and quantitative research components of a study relate to each other. These purposes can be used post hoc to classify research or a priori in the design of a new study. When designing a mixed methods study, it is sometimes helpful to list the purpose in the title of the study design.

The key point of this section is for the researcher to begin a study with at least one research question and then carefully consider what the purposes for mixing are. One can use mixed methods to examine different aspects of a single research question, or one can use separate but related qualitative and quantitative research questions. In all cases, the mixing of methods, methodologies, and/or paradigms will help answer the research questions and make improvements over a more basic study design. Fuller and richer information will be obtained in the mixed methods study.

## Theoretical drive

In addition to a mixing purpose, a mixed methods research study might have an overall “theoretical drive” (Morse and Niehaus 2009). When designing a mixed methods study, it is *occasionally* helpful to list the theoretical drive in the title of the study design. An investigation, in Morse and Niehaus’s (2009) view, is focused primarily on either exploration-and-description or on testing-and-prediction. In the first case, the theoretical drive is called “inductive” or “qualitative”; in the second case, it is called “deductive” or “quantitative”. In the case of mixed methods, the component that corresponds to the theoretical drive is referred to as the “core” component (“Kernkomponente”), and the other component is called the “supplemental” component (“ergänzende Komponente”). In Morse’s notation system, the core component is written in capitals and the supplemental component is written in lowercase letters. For example, in a QUAL → quan design, more weight is attached to the data coming from the core qualitative component. Due to the decisive character of the core component, the core component must be able to stand on its own, and should be implemented rigorously. The supplemental component does not have to stand on its own.

Although this distinction is useful in some circumstances, we do not advise to apply it to every mixed methods design. First, Morse and Niehaus contend that the supplemental component can be done “less rigorously” but do not explain which aspects of rigor can be dropped. In addition, the idea of decreased rigor is in conflict with one key theme of the present article, namely that mixed methods designs should always meet the criterion of multiple validities legitimation (Onwuegbuzie and Johnson 2006).

The idea of theoretical drive as explicated by Morse and Niehaus has been criticized. For example, we view a theoretical drive as a feature not of a whole study, but of a research question, or, more precisely, of an interpretation of a research question. For example, if one study includes multiple research questions, it might include several theoretical drives (Schoonenboom 2016).

Another criticism of Morse and Niehaus’ conceptualization of theoretical drive is* that it does not allow for equal-status mixed methods research* (“Mixed Methods Forschung, bei der qualitative und quantitative Methoden die gleiche Bedeutung haben” or “gleichrangige Mixed Methods-Designs”), in which both the qualitative and quantitative component are of equal value and weight; this same criticism applies to Morgan’s (2014) set of designs. We agree with Greene (2015) that mixed methods research can be integrated at the levels of method, methodology, and paradigm. In this view, equal-status mixed methods research designs are possible, and they result when both the qualitative and the quantitative components, approaches, and thinking are of equal value, they take control over the research process in alternation, they are in constant interaction, and the outcomes they produce are integrated during and at the end of the research process. Therefore, equal-status mixed methods research (that we often advocate) is also called “interactive mixed methods research”.

Mixed methods research can have three different drives, as formulated by Johnson et al. (2007, p. 123):

Qualitative dominant [or qualitatively driven] mixed methods research is the type of mixed research in which one relies on a qualitative, constructivist-poststructuralist-critical view of the research process, while concurrently recognizing that the addition of quantitative data and approaches are likely to benefit most research projects. Quantitative dominant [or quantitatively driven] mixed methods research is the type of mixed research in which one relies on a quantitative, postpositivist view of the research process, while concurrently recognizing that the addition of qualitative data and approaches are likely to benefit most research projects. (p. 124)

The area around the center of the [qualitative-quantitative] continuum, *equal status*, is the home for the person that self-identifies as a mixed methods researcher. This researcher takes as his or her starting point the logic and philosophy of mixed methods research. These mixed methods researchers are likely to believe that qualitative and quantitative data and approaches will add insights as one considers most, if not all, research questions.

We leave it to the reader to decide if he or she desires to conduct a qualitatively driven study, a quantitatively driven study, or an equal-status/“interactive” study. According to the philosophies of pragmatism (Johnson and Onwuegbuzie 2004) and dialectical pluralism (Johnson 2017), interactive mixed methods research is very much a possibility. By successfully conducting an equal-status study, the pragmatist researcher shows that paradigms can be mixed or combined, and that the incompatibility thesis does not always apply to research practice. Equal status research is most easily conducted when a research team is composed of qualitative, quantitative, and mixed researchers, interacts continually, and conducts a study to address one superordinate goal.

## Timing: simultaneity and dependence

Another important distinction when designing a mixed methods study relates to the timing of the two (or more) components. When designing a mixed methods study, it is usually helpful to include the word “concurrent” (“parallel”) or “sequential” (“sequenziell”) in the title of the study design; a complex design can be partially concurrent and partially sequential. Timing has two aspects: simultaneity and dependence (Guest 2013).

*Simultaneity (“Simultanität”)* forms the basis of the distinction between concurrent and sequential designs. In a *sequential design*, the quantitative component precedes the qualitative component, or vice versa. In a *concurrent design*, both components are executed (almost) simultaneously. In the notation of Morse (1991), concurrence is indicated by a “+” between components (e. g., QUAL + quan), while sequentiality is indicated with a “→” (QUAL → quan). Note that the use of capital letters for one component and lower case letters for another component in the same design suggest that one component is primary and the other is secondary or supplemental.

Some designs are sequential by nature. For example, in a *conversion design,* qualitative categories and themes might be first obtained by collection and analysis of qualitative data, and then subsequently quantitized (Teddlie and Tashakkori 2009). Likewise, with Greene et al.’s (1989) initiation purpose, the initiation strand follows the unexpected results that it is supposed to explain. In other cases, the researcher has a choice. It is possible, e. g., to collect interview data and survey data of one inquiry simultaneously; in that case, the research activities would be concurrent. It is also possible to conduct the interviews after the survey data have been collected (or vice versa); in that case, research activities are performed sequentially. Similarly, a study with the purpose of expansion can be designed in which data on an effect and the intervention process are collected simultaneously, or they can be collected sequentially.

A second aspect of timing is *dependence (“Abhängigkeit”)*. We call two research components *dependent* if the implementation of the second component depends on the results of data analysis in the first component. Two research components are *independent*, if their implementation does not depend on the results of data analysis in the other component. Often, a researcher has a choice to perform data analysis independently or not. A researcher could analyze interview data and questionnaire data of one inquiry independently; in that case, the research activities would be independent. It is also possible to let the interview questions depend upon the outcomes of the analysis of the questionnaire data (or vice versa); in that case, research activities are performed dependently. Similarly, the empirical outcome/effect and process in a study with the purpose of expansion might be investigated independently, or the process study might take the effect/outcome as given (dependent).

In the mixed methods literature, the distinction between sequential and concurrent usually refers to the combination of concurrent/independent and sequential/dependent, and to the combination of data collection and data analysis. It is said that in a concurrent design, the data collection and data analysis of both components occurs (almost) simultaneously and independently, while in a sequential design, the data collection and data analysis of one component take place after the data collection and data analysis of the other component and depends on the outcomes of the other component.

In our opinion, simultaneity and dependence are two separate dimensions. Simultaneity indicates whether data *collection* is done concurrent or sequentially. Dependence indicates whether the implementation of one component depends upon the results of data* analysis* of the other component. As we will see in the example case studies, a concurrent design could include dependent data analysis, and a sequential design could include independent data analysis. It is conceivable that one simultaneously conducts interviews and collects questionnaire data (concurrent), while allowing the analysis focus of the interviews to depend on what emerges from the survey data (dependence).

Dependent research activities include a redirection of subsequent research inquiry. Using the outcomes of the first research component, the researcher decides what to do in the second component. Depending on the outcomes of the first research component, the researcher will do *something else* in the second component. If this is so, the research activities involved are said to be sequential-dependent, and any component preceded by another component should appropriately build on the previous component (see *sequential validity legitimation*; Johnson and Christensen 2017; Onwuegbuzie and Johnson 2006).

It is under the purposive discretion of the researcher to determine whether a concurrent-dependent design, a concurrent-independent design, a sequential-dependent design, or a sequential-dependent design is needed to answer a particular research question or set of research questions in a given situation.

## Point of integration

Each true mixed methods study has at least one “point of integration” – called the “point of interface” by Morse and Niehaus (2009) and Guest (2013) –, at which the qualitative and quantitative components are brought together. Having one or more points of integration is the distinguishing feature of a design based on multiple components. It is at this point that the components are “mixed”, hence the label “mixed methods designs”. The term “mixing”, however, is misleading, as the components are not simply mixed, but have to be integrated very carefully.

Determining where the point of integration will be, and how the results will be integrated, is an important, if not the most important, decision in the design of mixed methods research. Morse and Niehaus (2009) identify two possible points of integration: the results point of integration and the analytical point of integration.

Most commonly, integration takes place in the *results point of integration*. At some point in writing down the results of the first component, the results of the second component are added and integrated. A *joint display* (listing the qualitative and quantitative findings and an integrative statement) might be used to facilitate this process.

In the case of an *analytical point of integration*, a first analytical stage of a qualitative component is followed by a second analytical stage, in which the topics identified in the first analytical stage are quantitized. The results of the qualitative component ultimately, and before writing down the results of the analytical phase as a whole, become quantitative; qualitizing also is a possible strategy, which would be the converse of this.

Other authors assume more than two possible points of integration. Teddlie and Tashakkori (2009) distinguish four different stages of an investigation: the conceptualization stage, the methodological experimental stage (data collection), the analytical experimental stage (data analysis), and the inferential stage. According to these authors, in all four stages, mixing is possible, and thus all four stages are potential points or integration.

However, the four possible points of integration used by Teddlie and Tashakkori (2009) are still too coarse to distinguish some types of mixing. Mixing in the experiential stage can take many different forms, for example the use of cognitive interviews to improve a questionnaire (tool development), or selecting people for an interview on the basis of the results of a questionnaire (sampling). Extending the definition by Guest (2013), we define the point of integration as “any point in a study where two or more research components are mixed or connected in some way”. Then, the point of integration in the two examples of this paragraph can be defined more accurately as “instrument development”, and “development of the sample”.

It is at the point of integration that qualitative and quantitative components are integrated. Some primary ways that the components can be connected to each other are as follows:

(1) merging the two data sets,

(2) connecting from the analysis of one set of data to the collection of a second set of data,

(3) embedding of one form of data within a larger design or procedure, and

(4) using a framework (theoretical or program) to bind together the data sets (Creswell and Plano Clark 2011, p. 76).

More generally, one can consider mixing at any or all of the following research components: purposes, research questions, theoretical drive, methods, methodology, paradigm, data, analysis, and results. One can also include mixing views of different researchers, participants, or stakeholders. The creativity of the mixed methods researcher designing a study is extensive.

Substantively, it can be useful to think of integration or mixing as comparing and bringing together two (or more) components on the basis of one or more of the purposes set out in the first section of this article. For example, it is possible to use qualitative data to *illustrate* a quantitative effect, or to determine whether the qualitative and the quantitative component yield convergent results (*triangulation*). An integrated result could also consist of a combination of a quantitatively established *effect* and a qualitative description of the underlying *process*. In the case of development, integration consists of an adjustment of an, often quantitative, for example, instrument or model or interpretation, based on qualitative assessments by members of the target group.

A special case is the integration of divergent results. The power of mixed methods research is its ability to deal with diversity and divergence. In the literature, we find two kinds of strategies for dealing with divergent results. A first set of strategies takes the detected divergence as the starting point for further analysis, with the aim to resolve the divergence. One possibility is to carry out further research (Cook 1985; Greene and Hall 2010). Further research is not always necessary. One can also look for a more comprehensive theory, which is able to account for both the results of the first component and the deviating results of the second component. This is a form of abduction (Erzberger and Prein 1997).

A fruitful starting point in trying to resolve divergence through abduction is to determine which component has resulted in a finding that is somehow expected, logical, and/or in line with existing research. The results of this research component, called the “sense” (“Lesart”), are subsequently compared to the results of the other component, called the “anti-sense” (“alternative Lesart”), which are considered dissonant, unexpected, and/or contrary to what had been found in the literature. The aim is to develop an overall explanation that fits both the sense and the anti-sense (Bazeley and Kemp 2012; Mendlinger and Cwikel 2008). Finally, a reanalysis of the data can sometimes lead to resolving divergence (Creswell and Plano Clark 2011).

Alternatively, one can question the existence of the encountered divergence. In this regard, Mathison (1988) recommends determining whether deviating results shown by the data can be explained by knowledge about the research and/or knowledge of the social world. Differences between results from different data sources could also be the result of properties of the methods involved, rather than reflect differences in reality (Yanchar and Williams 2006). In general, the conclusions of the individual components can be subjected to an inference quality audit (Teddlie and Tashakkori 2009), in which the researcher investigates the strength of each of the divergent conclusions. We recommend that researchers first determine whether there is “real” divergence, according to the strategies mentioned in the last paragraph. Next, an attempt can be made to resolve cases of “true” divergence, using one or more of the methods mentioned in this paragraph.

## Design typology utilization

As already mentioned in Sect. 1, mixed methods designs can be classified into a mixed methods typology or taxonomy. A typology serves several purposes, including the following: guiding practice, legitimizing the field, generating new possibilities, and serving as a useful pedagogical tool (Teddlie and Tashakkori 2009). Note, however, that not all types of typologies are equally suitable for all purposes. For generating new possibilities, one will need a more exhaustive typology, while a useful pedagogical tool might be better served by a non-exhaustive overview of the most common mixed methods designs. Although some of the current MM design typologies include more designs than others, none of the current typologies is fully exhaustive. When designing a mixed methods study, it is often useful to borrow its name from an existing typology, or to construct a superior and nuanced clear name when your design is based on a modification of one or more of the designs.

Various typologies of mixed methods designs have been proposed. Creswell and Plano Clark’s (2011) typology of some “commonly used designs” includes six “major mixed methods designs”. Our summary of these designs runs as follows:

- Convergent parallel design (“paralleles Design”) (the quantitative and qualitative strands of the research are performed independently, and their results are brought together in the overall interpretation),
- Explanatory sequential design (“explanatives Design”) (a first phase of quantitative data collection and analysis is followed by the collection of qualitative data, which are used to explain the initial quantitative results),
- Exploratory sequential design (“exploratives Design”) (a first phase of qualitative data collection and analysis is followed by the collection of quantitative data to test or generalize the initial qualitative results),
- Embedded design (“Einbettungs-Design”) (in a traditional qualitative or quantitative design, a strand of the other type is added to enhance the overall design),
- Transformative design (“politisch-transformatives Design”) (a transformative theoretical framework, e. g. feminism or critical race theory, shapes the interaction, priority, timing and mixing of the qualitative and quantitative strand),
- Multiphase design (“Mehrphasen-Design”) (more than two phases or both sequential and concurrent strands are combined over a period of time within a program of study addressing an overall program objective).

Most of their designs presuppose a specific juxtaposition of the qualitative and quantitative component. Note that the last design is a complex type that is required in many mixed methods studies.

The following are our adapted definitions of Teddlie and Tashakkori’s (2009) five *sets* of mixed methods research designs (adapted from Teddlie and Tashakkori 2009, p. 151):

- Parallel mixed designs (“paralleles Mixed-Methods-Design”) – In these designs, one has two or more parallel quantitative and qualitative strands, either with some minimal time lapse or simultaneously; the strand results are integrated into meta-inferences after separate analysis are conducted; related QUAN and QUAL research questions are answered or aspects of the same mixed research question is addressed.
- Sequential mixed designs (“sequenzielles Mixed-Methods-Design”) – In these designs, QUAL and QUAN strands occur across chronological phases, and the procedures/questions from the later strand emerge/depend/build on on the previous strand; the research questions are interrelated and sometimes evolve during the study.
- Conversion mixed designs (“Transfer-Design” or “Konversionsdesign”) – In these parallel designs, mixing occurs when one type of data is transformed to the other type and then analyzed, and the additional findings are added to the results; this design answers related aspects of the same research question,
- Multilevel mixed designs (“Mehrebenen-Mixed-Methods-Design”) – In these parallel or sequential designs, mixing occurs across multiple levels of analysis, as QUAN and QUAL data are analyzed and integrated to answer related aspects of the same research question or related questions.
- Fully integrated mixed designs (“voll integriertes Mixed-Methods-Design”) – In these designs, mixing occurs in an interactive manner at all stages of the study. At each stage, one approach affects the formulation of the other, and multiple types of implementation processes can occur. For example, rather than including integration only at the findings/results stage, or only across phases in a sequential design, mixing might occur at the conceptualization stage, the methodological stage, the analysis stage, and the inferential stage.

We recommend adding to Teddlie and Tashakkori’s typology a sixth design type, specifically, a *“hybrid” design* type to include complex combinations of two or more of the other design types. We expect that many published MM designs will fall into the hybrid design type.

Morse and Niehaus (2009) listed eight mixed methods designs in their book (and suggested that authors create more complex combinations when needed). Our shorthand labels and descriptions (adapted from Morse and Niehaus 2009, p. 25) run as follows:

- QUAL + quan (inductive-simultaneous design where, the core component is qualitative and the supplemental component is quantitative)
- QUAL → quan (inductive-sequential design, where the core component is qualitative and the supplemental component is quantitative)
- QUAN + qual (deductive-simultaneous design where, the core component is quantitative and the supplemental component is qualitative)
- QUAN → qual (deductive-sequential design, where the core component is quantitative and the supplemental component is qualitative)
- QUAL + qual (inductive-simultaneous design, where both components are qualitative; this is a multimethod design rather than a mixed methods design)
- QUAL → qual (inductive-sequential design, where both components are qualitative; this is a multimethod design rather than a mixed methods design)
- QUAN + quan (deductive-simultaneous design, where both components are quantitative; this is a multimethod design rather than a mixed methods design)
- QUAN → quan (deductive-sequential design, where both components are quantitative; this is a multimethod design rather than a mixed methods design).

Notice that Morse and Niehaus (2009) included four mixed methods designs (the first four designs shown above) and four multimethod designs (the second set of four designs shown above) in their typology. The reader can, therefore, see that the design notation also works quite well for multimethod research designs. Notably absent from Morse and Niehaus’s book are equal-status or interactive designs. In addition, they assume that the core component should always be performed either concurrent with or before the supplemental component.

Johnson, Christensen, and Onwuegbuzie constructed a set of mixed methods designs without these limitations. The resulting mixed methods design matrix (see Johnson and Christensen 2017, p. 478) contains nine designs, which we can label as follows (adapted from Johnson and Christensen 2017, p. 478):

- QUAL + QUAN (equal-status concurrent design),
- QUAL + quan (qualitatively driven concurrent design),
- QUAN + qual (quantitatively driven concurrent design),
- QUAL → QUAN (equal-status sequential design),
- QUAN → QUAL (equal-status sequential design),
- QUAL → quan (qualitatively driven sequential design),
- qual → QUAN (quantitatively driven sequential design),
- QUAN → qual (quantitatively driven sequential design), and
- quan → QUAL (qualitatively driven sequential design).

The above set of nine designs assumed only one qualitative and one quantitative component. However, this simplistic assumption can be relaxed in practice, allowing the reader to construct more complex designs. The Morse notation system is very powerful. For example, here is a three-stage equal-status concurrent-sequential design:

$$\left (\text{QUAL }+\text{ QUAN}\right )\rightarrow \text{ QUAN }\rightarrow \text{ QUAL}$$

The key point here is that the Morse notation provides researchers with a powerful language for depicting and communicating the design constructed for a specific research study.

When designing a mixed methods study, it is sometimes helpful to include the mixing purpose (or characteristic on one of the other dimensions shown in Table 1) in the title of the study design (e. g., an explanatory sequential MM design, an exploratory-confirmatory MM design, a developmental MM design). Much more important, however, than a design name is for the author to provide an accurate description of what was done in the research study, so the reader will know exactly how the study was conducted. A design classification label can never replace such a description.

The common complexity of mixed methods design poses a problem to the above typologies of mixed methods research. The typologies were designed to classify whole mixed methods studies, and they are basically based on a classification of simple designs. In practice, many/most designs are complex. Complex designs are sometimes labeled “complex design”, “multiphase design”, “fully integrated design”, “hybrid design” and the like. Because complex designs occur very often in practice, the above typologies are not able to classify a large part of existing mixed methods research any further than by labeling them “complex”, which in itself is not very informative about the particular design. This problem does not fully apply to Morse’s notation system, which can be used to symbolize some more complex designs.

Something similar applies to the classification of the purposes of mixed methods research. The classifications of purposes mentioned in the “Purpose”-section, again, are basically meant for the classification of whole mixed methods studies. In practice, however, one single study often serves more than one purpose (Schoonenboom et al. 2017). The more purposes that are included in one study, the more difficult it becomes to select a design on the basis of the purpose of the investigation, as advised by Greene (2007). Of all purposes involved, then, which one should be the primary basis for the design? Or should the design be based upon all purposes included? And if so, how? For more information on how to articulate design complexity based on multiple purposes of mixing, see Schoonenboom et al. (2017).

It should be clear to the reader that, although much progress has been made in the area of mixed methods design typologies, the problem remains in developing a single typology that is effective in comprehensively listing a set of designs for mixed methods research. This is why we emphasize in this article the importance of learning to build on simple designs and *construct* one’s own design for one’s research questions. This will often result in a combination or “hybrid” design that goes beyond basic designs found in typologies, and a methodology section that provides much more information than a design name.

## Typological versus interactive approaches to design

In the introduction, we made a distinction between design as a product and design as a process. Related to this, two different approaches to design can be distinguished: typological/taxonomic approaches (“systematische Ansätze”), such as those in the previous section, and interactive approaches (“interaktive Ansätze”) (the latter were called “dynamic” approaches by Creswell and Plano Clark 2011). Whereas typological/taxonomic approaches view designs as a sort of mold, in which the inquiry can be fit, interactive approaches (Maxwell 2013) view design as a process, in which a certain design-as-a-product might be the outcome of the process, but not its input.

The most frequently mentioned *interactive approach* to mixed methods research is the approach by Maxwell and Loomis (2003). Maxwell and Loomis distinguish the following components of a design: goals, conceptual framework, research question, methods, and validity. They argue convincingly that the most important task of the researcher is to deliver as the end product of the design process a design in which these five components fit together properly. During the design process, the researcher works alternately on the individual components, and as a result, their initial fit, if it existed, tends to get lost. The researcher should therefore regularly check during the research and continuing design process whether the components still fit together, and, if not, should adapt one or the other component to restore the fit between them. In an interactive approach, unlike the typological approach, design is viewed as an interactive process in which the components are continually compared during the research study to each other and adapted to each other.

Typological and interactive approaches to mixed methods research have been presented as mutually exclusive alternatives. In our view, however, they are not mutually exclusive. The interactive approach of Maxwell is a very powerful tool for conducting research, yet this approach is not specific to mixed methods research. Maxwell’s interactive approach emphasizes that the researcher should keep and monitor a close fit between the five components of research design. However, it does not indicate how one should combine qualitative and quantitative subcomponents within one of Maxwell’s five components (e. g., how one should combine a qualitative and a quantitative method, or a qualitative and a quantitative research question). Essential elements of the design process, such as timing and the point of integration are not covered by Maxwell’s approach. This is not a shortcoming of Maxwell’s approach, but it indicates that to support the design of mixed methods research, more is needed than Maxwell’s model currently has to offer.

Some authors state that design typologies are particularly useful for beginning researchers and interactive approaches are suited for experienced researchers (Creswell and Plano Clark 2011). However, like an experienced researcher, a research novice needs to align the components of his or her design properly with each other, and, like a beginning researcher, an advanced researcher should indicate how qualitative and quantitative components are combined with each other. This makes an interactive approach desirable, also for beginning researchers.

We see two merits of the *typological/taxonomic approach*. We agree with Greene (2007), who states that the value of the typological approach mainly lies in the different dimensions of mixed methods that result from its classifications. In this article, the primary dimensions include purpose, theoretical drive, timing, point of integration, typological vs. interactive approaches, planned vs. emergent designs, and complexity (also see secondary dimensions in Table 1). Unfortunately, all of these dimensions are not reflected in any single design typology reviewed here. A second merit of the typological approach is the provision of common mixed methods research designs, of common ways in which qualitative and quantitative research can be combined, as is done for example in the major designs of Creswell and Plano Clark (2011). Contrary to other authors, however, we do not consider these designs as a feature of a whole study, but rather, in line with Guest (2013), as a feature of one part of a design in which one qualitative and one quantitative component are combined. Although one study could have only one purpose, one point of integration, et cetera, we believe that combining “designs” is the rule and not the exception. Therefore, complex designs need to be constructed and modified as needed, and during the writing phase the design should be described in detail and perhaps given a creative and descriptive name.

## Planned versus emergent designs

A mixed methods design can be thought out in advance, but can also arise during the course of the conduct of the study; the latter is called an “emergent” design (Creswell and Plano Clark 2011). Emergent designs arise, for example, when the researcher discovers during the study that one of the components is inadequate (Morse and Niehaus 2009). Addition of a component of the other type can sometimes remedy such an inadequacy. Some designs contain an emergent component by their nature. Initiation, for example, is the further exploration of unexpected outcomes. Unexpected outcomes are by definition not foreseen, and therefore cannot be included in the design in advance.

The question arises whether researchers should plan all these decisions beforehand, or whether they can make them during, and depending on the course of, the research process. The answer to this question is twofold. On the one hand, a researcher should decide beforehand which research components to include in the design, such that the conclusion that will be drawn will be robust. On the other hand, developments during research execution will sometimes prompt the researcher to decide to add additional components. In general, the advice is to be prepared for the unexpected. When one is able to plan for emergence, one should not refrain from doing so.

## Dimension of complexity

Next, mixed methods designs are characterized by their complexity. In the literature, simple and complex designs are distinguished in various ways. A common distinction is between simple investigations with a single point of integration versus complex investigations with multiple points of integration (Guest 2013). When designing a mixed methods study, it can be useful to mention in the title whether the design of the study is simple or complex. The primary message of this section is as follows: *It is the responsibility of the researcher to create more complex designs when needed to answer his or her research question(s)*.

Teddlie and Tashakkori’s (2009) multilevel mixed designs and fully integrated mixed designs are both complex designs, but for different reasons. A multilevel mixed design is more complex ontologically, because it involves multiple levels of reality. For example, data might be collected both at the levels of schools and students, neighborhood and households, companies and employees, communities and inhabitants, or medical practices and patients (Yin 2013). Integration of these data does not only involve the integration of qualitative and quantitative data, but also the integration of data originating from different sources and existing at different levels. Little if any published research has discussed the possible ways of integrating data obtained in a multilevel mixed design (see Schoonenboom 2016). This is an area in need of additional research.

The fully-integrated mixed design is more complex because it contains multiple points of integration. As formulated by Teddlie and Tashakkori (2009, p. 151):

In these designs, mixing occurs in an interactive manner at all stages of the study. At each stage, one approach affects the formulation of the other, and multiple types of implementation processes can occur.

Complexity, then, not only depends on the number of components, but also on the extent to which they depend on each other (e. g., “one approach *affects* the formulation of the other”).

Many of our design dimensions ultimately refer to different ways in which the qualitative and quantitative research components are interdependent. Different purposes of mixing ultimately differ in the way one component relates to, and depends upon, the other component. For example, these purposes include dependencies, such as “x illustrates y” and “x explains y”. Dependencies in the implementation of x and y occur to the extent that the design of y depends on the results of x (sequentiality). The theoretical drive creates dependencies, because the supplemental component y is performed and interpreted within the context and the theoretical drive of core component x. As a general rule in designing mixed methods research, one should examine and plan carefully the ways in which and the extent to which the various components depend on each other.

The dependence among components, which may or may not be present, has been summarized by Greene (2007). It is seen in the distinction between *component designs* (“Komponenten-Designs”), in which the components are independent of each other, and *integrated designs *(“integrierte Designs”), in which the components are interdependent. Of these two design categories, integrated designs are the more complex designs.

## Secondary design considerations

The primary design dimensions explained above have been the focus of this article. There are a number of secondary considerations for researchers to also think about when they design their studies (Johnson and Christensen 2017). Now we list some secondary design issues and questions that should be thoughtfully considered during the construction of a strong mixed methods research design.

- *Phenomenon:* Will the study be addressing (a) the same part or different parts of one phenomenon? (b) different phenomena?, or (c) the phenomenon/phenomena from different perspectives? Is the phenomenon (a) expected to be unique (e. g., historical event, particular group)?, (b) something expected to be part of a more regular and predictable phenomenon, or (c) a complex mixture of these?
- *Social scientific theory:* Will the study generate a new substantive theory, test an already constructed theory, or achieve both in a sequential arrangement? Or is the researcher not interested in substantive theory based on empirical data?
- *Ideological drive:* Will the study have an explicitly articulated ideological drive (e. g., feminism, critical race paradigm, transformative paradigm)?
- *Combination of sampling methods:* What specific quantitative sampling method(s) will be used? What specific qualitative sampling methods(s) will be used? How will these be combined or related?
- *Degree to which the research participants will be similar or different:* For example, participants or stakeholders with known differences of perspective would provide participants that are quite different.
- *Degree to which the researchers on the research team will be similar or different:* For example, an experiment conducted by one researcher would be high on similarity, but the use of a heterogeneous and participatory research team would include many differences.
- *Implementation setting:* Will the phenomenon be studied naturalistically, experimentally, or through a combination of these?
- *Degree to which the methods similar or different:* For example, a structured interview and questionnaire are fairly similar but administration of a standardized test and participant observation in the field are quite different.
- *Validity criteria and strategies: *What validity criteria and strategies will be used to address the defensibility of the study and the conclusions that will be drawn from it (see Chapter 11 in Johnson and Christensen 2017)?
- *Full study:* Will there be essentially one research study or more than one? How will the research report be structured?

## Two case studies

The above design dimensions are now illustrated by examples. A nice collection of examples of mixed methods studies can be found in Hesse-Biber (2010), from which the following examples are taken. The description of the first case example is shown in Box 1.

### Box 1

*Summary of Roth* (2006), *research regarding the gender-wage gap within Wall Street securities firms. Adapted from Hesse-Biber* (2010, pp. 457–458)

Louise Marie Roth’s research, *Selling Women Short: Gender and Money on Wall Street *(2006), tackles gender inequality in the workplace. She was interested in understanding the gender-wage gap among highly performing Wall Street MBAs, who on the surface appeared to have the same “human capital” qualifications and were placed in high-ranking Wall Street securities firms as their first jobs. In addition, Roth wanted to understand the “structural factors” within the workplace setting that may contribute to the gender-wage gap and its persistence over time. […] Roth conducted semistructured interviews, nesting quantitative closed-ended questions into primarily qualitative in-depth interviews […] In analyzing the quantitative data from her sample, she statistically considered all those factors that might legitimately account for gendered differences such as number of hours worked, any human capital differences, and so on. Her analysis of the quantitative data revealed the presence of a significant gender gap in wages that remained unexplained after controlling for any legitimate factors that might otherwise make a difference. […] Quantitative findings showed the extent of the wage gap while providing numerical understanding of the disparity but did not provide her with an understanding of the specific processes within the workplace that might have contributed to the gender gap in wages. […] Her respondents’ lived experiences over time revealed the hidden inner structures of the workplace that consist of discriminatory organizational practices with regard to decision making in performance evaluations that are tightly tied to wage increases and promotion.

This example nicely illustrates the distinction we made between simultaneity and dependency. On the two aspects of the timing dimension, this study was a concurrent-dependent design answering a set of related research questions. The data collection in this example was conducted simultaneously, and was thus concurrent – the quantitative closed-ended questions were embedded into the qualitative in-depth interviews. In contrast, the analysis was dependent, as explained in the next paragraph.

One of the purposes of this study was explanation: The qualitative data were used to understand the processes underlying the quantitative outcomes. It is therefore an explanatory design, and might be labelled an “explanatory concurrent design”. Conceptually, explanatory designs are often dependent: The qualitative component is used to explain and clarify the outcomes of the quantitative component. In that sense, the qualitative analysis in the case study took the outcomes of the quantitative component (“the existence of the gender-wage gap” and “numerical understanding of the disparity”), and aimed at providing an explanation for *that result of the quantitative data analysis*, by relating it to the contextual circumstances in which the quantitative outcomes were produced. This purpose of mixing in the example corresponds to Bryman’s (2006) “contextual understanding”. On the other primary dimensions, (a) the design was ongoing over a three-year period but was not emergent, (b) the point of integration was results, and (c) the design was not complex with respect to the point of integration, as it had only one point of integration. Yet, it was complex in the sense of involving multiple levels; both the level of the individual and the organization were included. According to the approach of Johnson and Christensen (2017), this was a QUAL + quan design (that was qualitatively driven, explanatory, and concurrent). If we give this study design a name, perhaps it should focus on what was done in the study: “explaining an effect from the process by which it is produced”. Having said this, the name “explanatory concurrent design” could also be used.

The description of the second case example is shown in Box 2.

### Box 2

*Summary of McMahon’s* (2007) *explorative study of the meaning, role, and salience of rape myths within the subculture of college student athletes. Adapted from Hesse-Biber* (2010, pp. 461–462)

Sarah McMahon (2007) wanted to explore the subculture of college student athletes and specifically the meaning, role, and salience of rape myths within that culture. […] While she was looking for confirmation between the quantitative ([structured] survey) and qualitative (focus groups and individual interviews) findings, she entered this study skeptical of whether or not her quantitative and qualitative findings would mesh with one another. McMahon […] first administered a survey [instrument] to 205 sophomore and junior student athletes at one Northeast public university. […] The quantitative data revealed a very low acceptance of rape myths among this student population but revealed a higher acceptance of violence among men and individuals who did not know a survivor of sexual assault. In the second qualitative (QUAL) phase, “focus groups were conducted as semi-structured interviews” and facilitated by someone of the same gender as the participants (p. 360). […] She followed this up with a third qualitative component (QUAL), individual interviews, which were conducted to elaborate on themes discovered in the focus groups and determine any differences in students’ responses between situations (i. e., group setting vs. individual). The interview guide was designed specifically to address focus group topics that needed “more in-depth exploration” or clarification (p. 361). The qualitative findings from the focus groups and individual qualitative interviews revealed “subtle yet pervasive rape myths” that fell into four major themes: “the misunderstanding of consent, the belief in ‘accidental’ and fabricated rape, the contention that some women provoke rape, and the invulnerability of female athletes” (p. 363). She found that the survey’s finding of a “low acceptance of rape myths … was contradicted by the findings of the focus groups and individual interviews, which indicated the presence of subtle rape myths” (p. 362).

On the timing dimension, this is an example of a sequential-independent design. It is sequential, because the qualitative focus groups were conducted after the survey was administered. The analysis of the quantitative and qualitative data was independent: Both were analyzed independently, to see whether they yielded the same results (which they did not). This purpose, therefore, was triangulation. On the other primary dimensions, (a) the design was planned, (b) the point of integration was results, and (c) the design was not complex as it had only one point of integration, and involved only the level of the individual. The author called this a “sequential explanatory” design. We doubt, however, whether this is the most appropriate label, because the qualitative component did not provide an explanation for quantitative results that were taken as given. On the contrary, the qualitative results contradicted the quantitative results. Thus, a “sequential-independent” design, or a “sequential-triangulation” design or a “sequential-comparative” design would probably be a better name.

Notice further that the second case study had the same point of integration as the first case study. The two components were brought together in the results. Thus, although the case studies are very dissimilar in many respects, this does not become visible in their point of integration. It can therefore be helpful to determine whether their point of extension is different. A *point of extension* is the point in the research process at which the second (or later) component comes into play. In the first case study, two related, but different research questions were answered, namely the quantitative question “How large is the gender-wage gap among highly performing Wall Street MBAs after controlling for any legitimate factors that might otherwise make a difference?”, and the qualitative research question “How do structural factors within the workplace setting contribute to the gender-wage gap and its persistence over time?” This case study contains one qualitative research question and one quantitative research question. Therefore, the point of extension is the research question. In the second case study, both components answered the same research question. They differed in their data collection (and subsequently in their data analysis): qualitative focus groups and individual interviews versus a quantitative questionnaire. In this case study, the point of extension was data collection. Thus, the point of extension can be used to distinguish between the two case studies.

## Summary and conclusions

The purpose of this article is to help researchers to understand how to design a mixed methods research study. Perhaps the simplest approach is to design is to look at a single book and select one from the few designs included in that book. We believe that is only useful as a starting point. Here we have shown that one often needs to *construct* a research design to fit one’s unique research situation and questions.

First, we showed that there are there are many *purposes* for which qualitative and quantitative methods, methodologies, and paradigms can be mixed. This must be determined in interaction with the research questions. Inclusion of a purpose in the design name can sometimes provide readers with useful information about the study design, as in, e. g., an “explanatory sequential design” or an “exploratory-confirmatory design”.

The second dimension is *theoretical drive* in the sense that Morse and Niehaus (2009) use this term. That is, will the study have an inductive or a deductive drive, or, we added, a combination of these. Related to this idea is whether one will conduct a qualitatively driven, a quantitatively driven, or an equal-status mixed methods study. This language is sometimes included in the design name to communicate this characteristic of the study design (e. g., a “quantitatively driven sequential mixed methods design”).

The third dimension is *timing*, which has two aspects: simultaneity and dependence. *Simultaneity* refers to whether the components are to be implemented concurrently, sequentially, or a combination of these in a multiphase design. Simultaneity is commonly used in the naming of a mixed methods design because it communicates key information. The second aspect of timing, *dependence*, refers to whether a later component depends on the results of an earlier component, e. g., Did phase two specifically build on phase one in the research study? The fourth design dimension is the *point of integration,* which is where the qualitative and quantitative components are brought together and integrated. This is an essential dimension, but it usually does not need to be incorporated into the design name.

The fifth design dimension is that of *typological vs. interactive design approaches*. That is, will one select a design from a typology or use a more interactive approach to construct one’s own design? There are many typologies of designs currently in the literature. Our recommendation is that readers examine multiple design typologies to better understand the design process in mixed methods research and to understand what designs have been identified as popular in the field. However, when a design that would follow from one’s research questions is not available, the researcher can and should (a) combine designs into new designs or (b) simply construct a new and unique design. One can go a long way in depicting a complex design with Morse’s (1991) notation when used to its full potential. We also recommend that researchers understand the process approach to design from Maxwell and Loomis (2003), and realize that research design is a process and it needs, oftentimes, to be flexible and interactive.

The sixth design dimension or consideration is whether a design will be fully specified during the planning of the research study or if the design (or part of the design) will be allowed to *emerge* during the research process, or a combination of these. The seventh design dimension is called *complexity*. One sort of complexity mentioned was multilevel designs, but there are many complexities that can enter designs. The key point is that good research often requires the use of complex designs to answer one’s research questions. This is not something to avoid. It is the responsibility of the researcher to learn how to construct and describe and name mixed methods research designs. Always remember that designs should follow from one’s research questions and purposes, rather than questions and purposes following from a few currently named designs.

In addition to the six primary design dimensions or considerations, we provided a set of additional or *secondary dimensions/considerations* or questions to ask when constructing a mixed methods study design. Our purpose throughout this article has been to show what factors must be considered to design a high quality mixed methods research study. The more one knows and thinks about the primary and secondary dimensions of mixed methods design the better equipped one will be to pursue mixed methods research.

## Appendix

### Bryman’s (2006) scheme of rationales for combining quantitative and qualitative research^1^

- *Triangulation *or greater validity – refers to the traditional view that quantitative and qualitative research might be combined to triangulate findings in order that they may be mutually corroborated. If the term was used as a synonym for integrating quantitative and qualitative research, it was not coded as triangulation.
- *Offset *– refers to the suggestion that the research methods associated with both quantitative and qualitative research have their own strengths and weaknesses so that combining them allows the researcher to offset their weaknesses to draw on the strengths of both.
- *Completeness *– refers to the notion that the researcher can bring together a more comprehensive account of the area of enquiry in which he or she is interested if both quantitative and qualitative research are employed.
- *Process *– quantitative research provides an account of structures in social life but qualitative research provides sense of process.
- *Different research questions *– this is the argument that quantitative and qualitative research can each answer different research questions but this item was coded only if authors explicitly stated that they were doing this.
- *Explanation *– one is used to help explain findings generated by the other.
- *Unexpected results *– refers to the suggestion that quantitative and qualitative research can be fruitfully combined when one generates surprising results that can be understood by employing the other.
- *Instrument development *– refers to contexts in which qualitative research is employed to develop questionnaire and scale items – for example, so that better wording or more comprehensive closed answers can be generated.
- *Sampling *– refers to situations in which one approach is used to facilitate the sampling of respondents or cases.
- *Credibility *– refer s to suggestions that employing both approaches enhances the integrity of findings.
- *Context *– refers to cases in which the combination is rationalized in terms of qualitative research providing contextual understanding coupled with either generalizable, externally valid findings or broad relationships among variables uncovered through a survey.
- *Illustration *– refers to the use of qualitative data to illustrate quantitative findings, often referred to as putting “meat on the bones” of “dry” quantitative findings.
- *Utility *or improving the usefulness of findings – refers to a suggestion, which is more likely to be prominent among articles with an applied focus, that combining the two approaches will be more useful to practitioners and others.
- *Confirm and discover *– this entails using qualitative data to generate hypotheses and using quantitative research to test them within a single project.
- *Diversity of views *– this includes two slightly different rationales – namely, combining researchers’ and participants’ perspectives through quantitative and qualitative research respectively, and uncovering relationships between variables through quantitative research while also revealing meanings among research participants through qualitative research.
- *Enhancement *or building upon quantitative/qualitative findings – this entails a reference to making more of or augmenting either quantitative or qualitative findings by gathering data using a qualitative or quantitative research approach.
- Other/unclear.
- Not stated.
